# Chinese herbal medicine improves the treating outcomes in advanced non-small cell lung cancer patients treated with iodine-125 seed brachytherapy: a 5-year follow-up study

**DOI:** 10.3389/fmed.2025.1595640

**Published:** 2025-06-25

**Authors:** Linjun Li, Cheng Zhang, Jun Luo, Ruiqin Zhou, Guoqing Zhou, Qingchen Wu

**Affiliations:** ^1^Department of Cardiothoracic Surgery, The First Affiliated Hospital of Chongqing Medical University, Chongqing, China; ^2^Department of Traditional Chinese Medicine, The First Affiliated Hospital of Chongqing Medical University, Chongqing, China

**Keywords:** advanced non-small cell lung cancer, chemotherapy, Chinese herbal medicine, iodine-125 seed, survival analysis

## Abstract

Brachytherapy based on iodine-125 (I-125) is becoming one of the alternative treatment option for advanced non-small cell lung cancer (NSCLC). Chinese herbal medicine (CHM) combined with radiotherapy reduces the complications. In the current study, we attempted to assess the outcomes of treating strategies using CHM, chemotherapy or I-125. 182 patients who underwent I-125 seed implantation alone or in combination with chemotherapy or CHM treatment were enrolled in the current study. The clinical information of the patients were collected, and analyzed after a 5-year follow-up. The overall survival rates at 1, 2, 3, and 5 years were 81, 47, 28, and 20%, respectively, with a median survival time of 24.28 months. For patients receiving chemotherapy combined with I-125 seed brachytherapy, the survival rates were 89, 53, 35, and 29%, respectively. In contrast, those treated with CHM combined with I-125 seed brachytherapy had survival rates of 90, 63, 42, and 23%. Meanwhile, the survival rates for patients treated with ^125^I seed brachytherapy alone were 69, 32, 12, and 11%. Additionally, patients receiving CHM combined with I-125 seed brachytherapy treatment also showed less complications such as cough and vomit. CHM treatment demonstrated comparable efficacy and less complications to chemotherapy in managing advanced NSCLC under the treatment of I-125.

## Introduction

Lung cancer remains the leading cause of cancer-related mortality worldwide, with non-small cell lung cancer (NSCLC) accounting for approximately 85% of all cases (1). Despite advancements in early detection and treatment strategies, most NSCLC patients are diagnosed at advanced stages (III-IV), where curative surgical resection is no longer a viable option. The standard-of-care for these patients includes systemic chemotherapy, radiotherapy, targeted therapy, and immunotherapy, with varying degrees of efficacy and toxicity. Iodine-125 (I-125) seed brachytherapy, a localized form of radiation therapy, has emerged as a promising approach for managing advanced NSCLC, offering improved tumor control with minimal impact on surrounding healthy tissues (2–4). However, its effectiveness is significantly influenced by the concurrent use of systemic therapies, such as chemotherapy or traditional Chinese herbal medicine (CHM), necessitating further comparative evaluation.

Despite the benefits of systemic chemotherapy, it is often associated with severe side effects, including hematologic toxicity, gastrointestinal disturbances, and immune suppression. Platinum-based doublet chemotherapy (cisplatin or carboplatin combined with paclitaxel, docetaxel, gemcitabine, or vinorelbine) remains the mainstay of palliative treatment, yet its efficacy is limited by drug resistance and treatment-related toxicities. The median overall survival (OS) for patients receiving chemotherapy alone remains less than 18 months, with 5-year survival rates below 10% (5, 6). Given these limitations, there is an increasing interest in integrating complementary treatment strategies, such as I-125 seed brachytherapy, which delivers continuous low-dose-rate (LDR) radiation to tumors, potentially enhancing treatment efficacy while reducing systemic toxicity.

I-125 seed brachytherapy involves implanting radioactive seeds directly into the tumor, ensuring localized and sustained radiation exposure. This technique has demonstrated significant benefits in tumor volume reduction, local disease control, and prolonged survival in patients with advanced NSCLC (7, 8). Compared to conventional external beam radiotherapy (EBRT), I-125 seed brachytherapy allows for higher radiation doses within the tumor while sparing surrounding healthy tissue, reducing complications such as radiation pneumonitis and esophagitis (9). However, brachytherapy alone may not be sufficient to control tumor progression, highlighting the necessity of combination therapies.

The combination of I-125 seed brachytherapy with chemotherapy has been explored as a means to enhance treatment outcomes. Several studies and meta-analyses suggest that I-125 combined with chemotherapy significantly improves objective response rates (ORR), disease control rates (DCR), and OS compared to chemotherapy alone. However, chemotherapy-related toxicities remain a substantial concern, often leading to dose reductions or early treatment discontinuation (10). In contrast, Chinese herbal medicine (CHM) has been widely used in China and other East Asian countries as an adjunct therapy for cancer treatment (11). CHM formulations, composed of bioactive compounds such as Astragalus, Ginseng, and Scutellaria, have demonstrated anti-tumor, anti-inflammatory, and immunomodulatory properties (12). Several randomized controlled trials (RCTs) and systematic reviews suggest that CHM, when combined with conventional therapies, can improve survival outcomes, reduce chemotherapy-induced side effects, and enhance quality of life (13). CHM exerts its effects through multiple mechanisms, including induction of apoptosis, inhibition of tumor angiogenesis, regulation of immune responses, and modulation of the tumor microenvironment (14).

Despite the growing interest in integrating I-125 seed brachytherapy with either chemotherapy or CHM, there is limited direct comparative data evaluating the efficacy and safety of these combination therapies. Most studies focus on I-125 with chemotherapy, while CHM remains an underexplored yet promising alternative for patients who are unfit for chemotherapy or seeking complementary treatments. Thus, in the current study, we aim to compare the survival outcomes, treatment response rates, and toxicity profiles of NSCLC patients treated with I-125 seed brachytherapy alone, I-125 + chemotherapy, and I-125 + CHM over a 5-year follow-up period. By conducting a comprehensive evaluation of these treatment strategies, we seek to determine whether CHM can serve as a viable alternative to chemotherapy in combination with I-125 brachytherapy. The findings of this study may provide valuable insights into the optimization of treatment strategies for advanced NSCLC patients, particularly in developing personalized, cost-effective, and well-tolerated therapeutic approaches.

## Materials and methods

### Study design and setting

**FIGURE 1:**
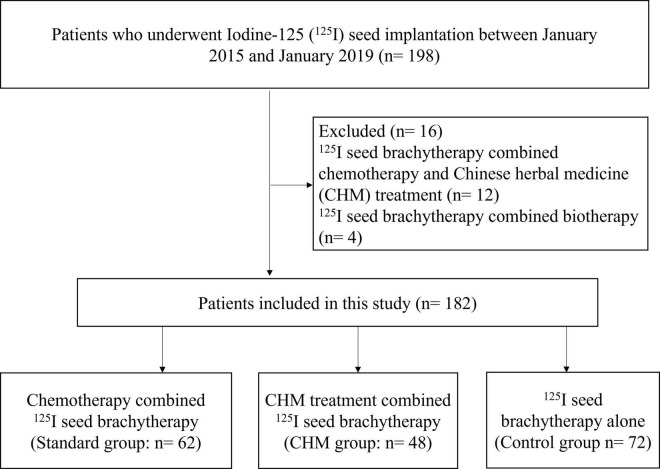
The flow chart of patient selection.

The current retrospectively enrolled 182 NCSCL patients treated with different strategies in The First Affiliated Hospital of Chongqing Medical University between January 2015 and January 2019, with a 5-year follow-up period until January 2024 (Figure 1). All the patients were selected based on clinical records and imaging studies. Preoperative staging of lung cancer primarily follows the 8th edition of the AJCC/UICC TNM staging system. Key diagnostic modalities for preoperative staging include: (1) Contrast-enhanced chest CT, which evaluates the size and location of the primary tumor and assesses lymph node involvement; (2) PET-CT, which detects systemic metabolic activity and identifies occult metastases; (3) Cranial CT, which helps rule out brain metastasis; (4) Bone scintigraphy/SPECT, used to screen for skeletal metastases; (5) Endobronchial ultrasound (EBUS), which accurately evaluates mediastinal lymph nodes (N staging); and (6) Percutaneous lung biopsy, which obtains tissue samples from deep-seated lesions.

The eligibility criteria were established to ensure that the study population was representative of individuals with advanced NSCLC receiving I-125 brachytherapy, either alone or in combination with chemotherapy or CHM: (1) Histopathologically confirmed Stage III NSCLC, deemed inoperable based on multidisciplinary team evaluation. (2) Received I-125 seed brachytherapy as part of their treatment plan, either as monotherapy or in combination with chemotherapy or CHM. (3) No prior systemic chemotherapy or radiotherapy before I-125 implantation, to ensure comparability among groups. (4) Age between 40 and 80 years, ensuring a focus on patients within a demographic range most likely to tolerate I-125 brachytherapy. (5) Eastern Cooperative Oncology Group (ECOG) performance status of 0–2, indicating that patients were functionally capable of undergoing treatment. (6) Life expectancy of at least 6 months at the time of treatment initiation. (7) Adequate organ function, defined by the following laboratory parameters: Hemoglobin (Hb) ≥ 9 g/dL, absolute neutrophil count (ANC) ≥ 1.5 × 10^9^/L, platelet count ≥ 100 × 10^9^/L, serum creatinine ≤ 1.5 × upper normal limit (UNL) or creatinine clearance ≥ 50 mL/min, alanine aminotransferase (ALT) and aspartate aminotransferase (AST) ≤ 2.5 × UNL, or ≤ 5 × UNL if liver metastases were present. (8) No known contraindications to I-125 brachytherapy, chemotherapy, or CHM, ensuring that patients could tolerate their respective treatments. (9) Ability and willingness to comply with scheduled follow-up visits, ensuring accurate data collection throughout the study period.

Patients were excluded from the study if they met any of the following conditions: (1) Stage IV NSCLC or presence of distant metastases, as the focus of this study was on locally advanced disease. (2) Tumor size > 7 cm, as larger tumors may require a different treatment strategy beyond brachytherapy. (3) Concurrent administration of both chemotherapy and CHM, as this would confound the comparative analysis of the two treatment combinations. (4) History of prior systemic therapy, including previous chemotherapy, radiotherapy, targeted therapy, or immunotherapy for NSCLC. (5) Severe comorbid conditions, including but not limited to: Uncontrolled cardiovascular disease, including severe heart failure (New York Heart Association Class III or IV) or unstable angina, severe pulmonary infections or active tuberculosis, which could interfere with lung function and response to treatment, severe hepatic or renal dysfunction, defined as ALT/AST > 5 × UNL, bilirubin > 3 × UNL or creatinine clearance < 30 mL/min, uncontrolled diabetes mellitus or active autoimmune diseases, which could affect treatment tolerance and survival outcomes, presence of another malignancy within the past 5 years, except for non-melanoma skin cancer or carcinoma *in situ* of the cervix, as previous cancers could confound survival outcomes, patients unable to complete follow-up assessments due to geographic limitations, cognitive impairment, or non-adherence to medical care, and known hypersensitivity to any chemotherapy agent or CHM component used in the study, ensuring patient safety. All I-125 seed implantation procedures and postoperative treatment protocols were performed following written informed consent obtained from both the patients and their legal guardians. The research protocol was formally approved by the Institutional Review Board. De-identified raw data supporting the findings have been archived in Supplementary Dataset. All the procedures were performed following the approval of ethic committee of The First Affiliated Hospital of Chongqing Medical University (Approval No. 2021-364) and the ethical standards in the 1964 Declaration of Helsinki and its later amendments.

### Group allocation and treatment

Patients were divided into three groups based on their treatment regimen: Control group (*n* = 72), I-125 Seed Brachytherapy Alone; Standard group (*n* = 62), I-125 Seed Brachytherapy + Chemotherapy; CHM group, I-125 Seed Brachytherapy + Chinese Herbal Medicine (*n* = 48). The detail treatment regime was as following.

#### I-125 Seed brachytherapy procedure

I-125 brachytherapy was performed using CT-guided percutaneous implantation of radioactive seeds, ensuring precise placement within the tumor. Preoperative treatment planning was conducted using a computerized treatment-planning system (TPS) to calculate tumor volume, radiation dose, and seed distribution. I-125 radioactive seeds (activity: 0.8 mCi per seed, half-life: 59.6 days) were implanted using 18G needles to achieve an optimized dose distribution. The total prescribed radiation dose ranged from 90–140 Gy, depending on tumor characteristics and seed placement density. The I-125 seeds used were 4.5 mm × 0.8 mm in size, with the needle spacing typically set at 1–1.5 cm, and the inter-particle spacing was 0.5 mm. Post-implantation dosimetry was performed via CT scan to verify seed distribution and ensure adequate tumor coverage. Patients were monitored for complications such as pneumonitis, local fibrosis, or seed migration following the procedure.

#### Chemotherapy regimen

Patients in the I-125 + chemotherapy group received platinum-based doublet chemotherapy, consisting of cisplatin (75 mg/m^2^ IV on day 1) combined with one of the following agents: Docetaxel (75 mg/m^2^ IV on day 1), vinorelbine (25 mg/m^2^ IV on days 1 and 8), gemcitabine (1,000 mg/m^2^ IV on days 1 and 8) or paclitaxel (175 mg/m^2^ IV on day 1). For lung adenocarcinoma, cisplatin in combination with either docetaxel or pemetrexed was typically administered, while cisplatin combined with paclitaxel was commonly used for squamous cell carcinoma. Chemotherapy cycles were repeated every 21 days for a total of 4–6 cycles, unless toxicity required dose adjustment or early discontinuation.

#### Chinese herbal medicine (CHM) regimen

Patients in the I-125 + CHM group received individualized oral CHM formulations for 6 months, starting within 1 week post-I-125 implantation. The anti-tumor herbal medicine formula was selected based on prescriptions from the Chinese Pharmacopeia and primarily includes the following herbs: Astragalus membranaceus (Huangqi) (active components: astragalus polysaccharides, astragalosides, flavonoids; dosage: 9–30 g), Panax ginseng (Renshen) (active components: ginsenosides, polysaccharides, amino acids; dosage: 6–10 g), Oldenlandia diffusa (Baihua She She Cao) (active components: glycoproteins, flavonoids, asperuloside; dosage: 15–30 g), Scutellaria baicalensis (Huangqin) (active components: baicalin, wogonoside, flavonoids; dosage: 6–10 g), and Glycyrrhiza uralensis (Gancao) (active components: glycyrrhizic acid, glycyrrhetinic acid, flavonoids; dosage: 2–10 g). The dosages of these herbs are adjusted based on the patient’s body weight, with higher doses prescribed for individuals with greater body weight. All herbs are decocted together in water and administered twice daily (morning and evening). The treatment regimen consists of 2–4 weeks per therapeutic cycle, with a total duration of 6 months.

### Follow-up and outcome measures

Patients underwent regular follow-up evaluations at every 3 months via telephone or outpatient clinic visits during the first year after implantation, and every 6 months thereafter. Primary endpoints included overall survival (OS), defined as time from treatment initiation to death. Six months after 125I seed implantation, CT scans were employed to measure lung tumor size. Follow-up consisted of medical history, clinical examination, and measurement of lung capacity. During the follow-up period, two patients were lost to follow-up despite multiple unsuccessful attempts to contact them. Cross-referencing with public health databases revealed no mortality records for these individuals. As a result, these patients were considered alive and included in the analysis in accordance with the intention-to-treat principle.

### Statistical analysis

Continuous variables were reported as means ± standard deviations (SD) or medians with interquartile ranges (IQRs) and analyzed using either two-sample *t*-tests for normally distributed data or Wilcoxon rank-sum tests for non-normally distributed data. Kaplan-Meier survival curves were generated to assess event-free survival based on categorical variables, and comparisons between curves were conducted using the Mantel-Cox log-rank test. Survival time was calculated from the date of local implantation of radioactive I-125 seeds until January 2024 or the time of death. All significant variables identified in the univariate analyses were included in a Cox proportional hazards regression model using a stepwise forward conditional method to determine independent prognostic factors. All statistical analyses were conducted using SPSS version 17.0 (SPSS Inc., Chicago, IL, United States), with *P* < 0.05 considered statistically significant.

## Results

### Baseline characteristics

A total of 182 patients diagnosed with Stage III NSCLC who underwent I-125 seed brachytherapy were included in this study. Patients were categorized into three groups based on their treatment regimen: I-125 seed brachytherapy alone (Control group, *n* = 72), I-125 seed brachytherapy combined with chemotherapy (Standard group, *n* = 62), and I-125 seed brachytherapy combined with CHM (CHM group, *n* = 48). The baseline characteristics, including age, gender, tumor site, tumor stage, tumor type, tumor size, and the number of I-125 seeds implanted, were well-balanced across the three groups, with no statistically significant differences observed (*P* > 0.05) (Table 1). For lung adenocarcinoma cases, the primary regimens consisted of cisplatin combined with either docetaxel or pemetrexed, while squamous cell carcinoma patients predominantly received cisplatin plus paclitaxel. Baseline characteristics revealed no statistically significant differences in squamous cell carcinoma versus adenocarcinoma distribution among the three groups (*P* = 0.668), suggesting minimal chemotherapy-specific selection bias (Table 1). The mean age of patients was 60.48 ± 8.01 years in the Standard group, 61.17 ± 9.76 years in the CHM group, and 59.40 ± 9.64 years in the Control group. Tumor distribution between the left and right lung was similar among groups, and the proportion of squamous cell carcinoma and adenocarcinoma did not show a statistically significant difference (*P* = 0.668). Additionally, preoperative complications, including hypertension, diabetes mellitus, and chronic obstructive pulmonary disease (COPD), were comparably distributed across the groups (*P* > 0.05) (Table 1).

**TABLE 1:** Baseline characteristics of the patients included in the study.

Characteristics	Standard group (*n* = 62)	CHM group (*n* = 48)	Control group (*n* = 72)	*P*-value
Gender				0.558
Male	57	42	62	
Female	5	6	10	
Age (years, mean ± SD)	60.48 ± 8.01	61.17 ± 9.76	59.40 ± 9.64	0.587
≤ 60	38	22	40	
>60	24	26	32	
Tumor site				0.902
Left lung	26	21	33	
Right lung	36	27	39	
Tumor staging				0.741
III A	30	22	38	
III B	32	26	34	
Tumor type				0.668
Squamous cell carcinoma	34	24	42	
Adenocarcinoma	28	24	30	
Tumor size				0.881
≤ 5 cm	37	27	40	
< 5 cm ≤ 7	25	21	32	
Number of ^125^I seeds (mean ± SD)	82.26 ± 21.46	78.63 ± 20.48	86.21 ± 19.70	0.163
**Preoperative complications**				
Hypertension	9	8	10	0.912
Diabetes mellitus	4	7	6	0.324
COPD	5	3	5	0.932

I-125, iodine-125; CHM, Chinese herbal medicine; SD, standard deviation; COPD, chronic obstructive pulmonary disease.

### Risk factors associated with mortality in all patients

The univariate Cox regression analysis identified chemotherapy and CHM treatment as significant protective factors against mortality, with hazard ratios (HR) of 0.487 (95% CI: 0.327–0.726, *P* = 0.000) for chemotherapy and 0.467 (95% CI: 0.304–0.717, *P* = 0.000) for CHM treatment, indicating improved survival in both treatment groups compared to I-125 seed brachytherapy alone. Other variables, including sex, tumor site, tumor size, and tumor type, did not significantly impact survival outcomes (*P* > 0.05) (Table 2). The multivariate Cox regression analysis further confirmed that both chemotherapy and CHM treatment significantly reduced mortality risk, with adjusted HR values of 0.506 (95% CI: 0.345–0.743, *P* = 0.001) for chemotherapy and 0.498 (95% CI: 0.331–0.750, *P* = 0.001) for CHM treatment, reaffirming their independent beneficial effects on survival (Table 3).

**TABLE 2:** Univariate Cox regression analysis for risk factors associated with mortality.

Variables	β	SE	Wald	df	sig	Exp (B)	95.0% CI for Exp (B)	
							Lower	Upper
Sex	−0.272	0.292	0.870	1	0.351	0.762	0.430	1.350
Chemotherapy	−0.761	0.219	12.118	1	0.000	0.467	0.304	0.717
CHM treatment	−0.719	0.203	12.531	1	0.000	0.487	0.327	0.726
Age	0.240	0.179	1.788	1	0.181	1.271	0.894	1.806
Tumor site	0.259	0.172	2.264	1	0.132	1.295	0.925	1.814
Tumor size	0.043	0.180	0.058	1	0.809	1.044	0.734	1.486
Tumor type	0.172	0.170	1.029	1	0.310	1.188	0.852	1.657
Tumor staging	0.090	0.183	0.244	1	0.622	1.094	0.765	1.566
Number of I-125 seeds	0.101	0.180	0.316	1	0.574	1.106	0.778	1.574

I-125, Iodine-125; CHM, Chinese herbal medicine.

**TABLE 3:** Multivariate Cox regression analysis for risk factors associated with mortality.

Variables	β	SE	Wald	df	sig	Exp (B)	95.0% CI for Exp (B)	
							Lower	Upper
Chemotherapy	−0.696	0.208	12.072	1	0.001	0.506	0.345	0.743
CHM treatment	−0.710	0.197	11.184	1	0.001	0.498	0.331	0.750

CHM, Chinese herbal medicine.

### Survival analysis

**FIGURE 2:**
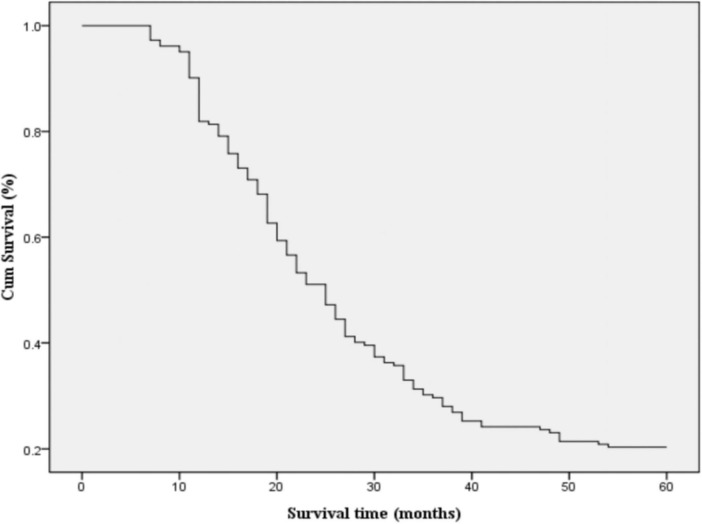
Overall survival of all included patients.

By the end of the follow-up period, a total of 159 deaths had occurred, corresponding to an incidence rate of 87.4%. Among these, 75 patients died from cachexia, 35 from multiple metastases, 30 from multiple organ failure, 16 from pulmonary infections, and 3 from massive hemoptysis. The overall survival rates at 1, 2, 3, and 5 years were 81, 47, 28, and 20%, respectively, with a median survival time of 24.28 months (Figure 2).

**FIGURE 3:**
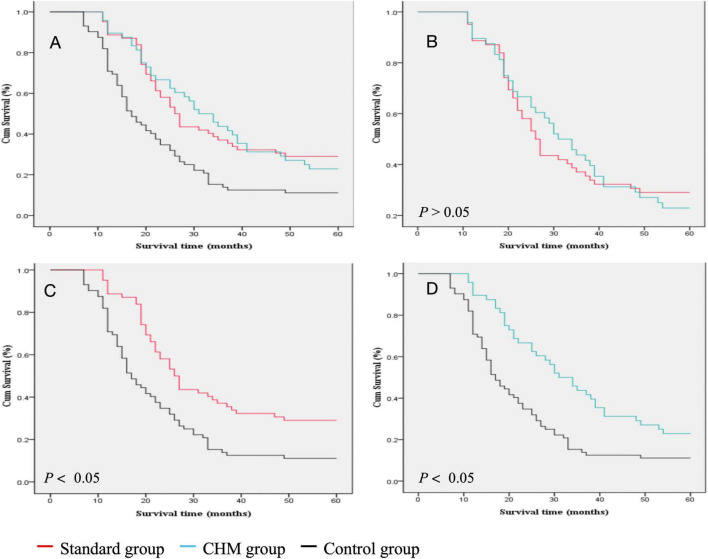
Comparison of the survival rate between different groups. **(A)** Survival rate comparison of all three groups. **(B)** Survival rate comparison of Standard group vs. CHM group. **(C)** survival rate comparison of survival rate of Standard group vs. Control group. **(D)** Survival rate comparison of survival rate of Control vs. CHM group.

Among the 182 patients, 62 received chemotherapy combined with I-125 seed brachytherapy, 48 received Chinese herbal medicine (CHM) treatment combined with I-125 seed brachytherapy, and 72 received I-125 seed brachytherapy alone. The 1-, 2-, 3-, and 5-year survival rates for patients treated with chemotherapy combined with I-125 seed brachytherapy were 89, 53, 35, and 29%, respectively. For those receiving CHM treatment combined with I-125 seed brachytherapy, the survival rates were 90, 63, 42, and 23%, respectively. Patients treated with I-125 seed brachytherapy alone had survival rates of 69, 32, 12, and 11% at 1, 2, 3, and 5 years, respectively. The median survival times for these three groups were 26.00, 31.00, and 16.5 months, respectively (Figure 3A).

There was no statistically significant difference in survival rates between patients treated with chemotherapy combined with I-125 seed brachytherapy (HR = 0.50, 95% CI [0.33, 0.75], *P* = 0.001) and those treated with CHM combined with I-125 seed brachytherapy (HR = 0.49, 95% CI [0.33, 0.72], *P* = 0.001) [Log-Rank (Mantel–Cox); χ^2^ = 0.002; *P* > 0.05]. The median survival times were 26.00 and 31.00 months, respectively (Figure 3B). However, the survival outcomes of chemotherapy combined with I-125 seed brachytherapy were significantly better than those of I-125 seed brachytherapy alone [Log-Rank (Mantel–Cox); χ^2^ = 12.784; *P* < 0.05], with median survival times of 26.00 and 16.50 months, respectively (Figure 3C). Similarly, CHM treatment combined with I-125 seed brachytherapy significantly prolonged survival compared to I-125 seed brachytherapy alone [Log-Rank (Mantel–Cox); χ^2^ = 11.687; *P* < 0.05], with median survival times of 31.00 and 16.50 months, respectively (Figure 3D). In the age stratification (≤ 60 years: 80 patients, > 60 years: 102 patients), subgroup analysis showed no statistically significant differences in prognostic survival between the age groups. Regarding cancer staging (Stage IIIA: 90 patients, Stage IIIB: 92 patients), no statistically significant difference in prognostic survival was observed between the two groups.

### Postoperative complications

Regarding postoperative complications, cough was most frequently reported in the Standard group (*n* = 39), followed by the CHM group (*n* = 13) and the Control group (*n* = 15), showing a statistically significant difference (*P* = 0.000). Similarly, vomiting occurred in 20 patients in the standard group, but only 2 patients in the CHM group and 3 in the Control group, indicating a significant difference (*P* = 0.000). However, there were no significant differences in the rates of pain, dyspnea, hemoptysis, or venous thrombosis among the groups (*P* > 0.05) (Table 4).

**TABLE 4:** Comparisons among the three groups concerning postoperative complications, causes of death, and treatment costs.

Variable	Standard group (*n* = 62)	CHM group (*n* = 48)	Control group (*n* = 72)	*P-*value
**Postoperative complications**				
Cough	39	13	15	0.000
Pain	14	10	17	0.938
Vomit	20	2	3	0.000
Dyspnea	3	2	7	0.384
Hemoptysis	2	4	6	0.421
Venous thrombosis	4	5	6	0.754
**Cause of death**				
Systemic metastasis	17	10	20	0.654
Hemoptysis	4	5	8	0.624
Respiratory failure	3	3	10	0.023
Treatment costs (mean ± SD) (Thousand)	95.8 ± 21.7	80.9 ± 18.2	57.1 ± 13.2	0.038

I-125, Iodine-125. CHM, Chinese herbal medicine. SD, Standard deviation.

Systemic metastasis was the most common cause of death across all groups, occurring in 17 patients in the Standard group, 10 in the CHM group, and 20 in the Control group (*P* = 0.654) (Table 4). Hemoptysis-related death was reported in 4, 5, and 8 patients in the Standard, CHM, and Control groups, respectively (*P* = 0.624) (Table 4). Notably, respiratory failure was significantly more frequent in the Control group (*n* = 10) compared to the Standard (*n* = 3) and CHM (*n* = 3) groups (*P* = 0.023) (Table 4), indicating that patients receiving either chemotherapy or CHM treatment had lower rates of respiratory failure-related mortality.

### Treatment costs

The economic burden of treatment varied significantly among the three groups. The mean treatment cost was highest in the Standard group (95.8 ± 21.7 thousand yuan), followed by the CHM group (80.9 ± 18.2 thousand yuan) and the Control group (57.1 ± 13.2 thousand yuan) (*P* = 0.038), suggesting that CHM therapy provided a cost-effective alternative to chemotherapy while still improving survival outcomes (Table 4).

## Discussion

The current study evaluated the long-term survival outcomes, treatment response rates, adverse events, and economic burden among patients with advanced NSCLC treated with I-125 seed brachytherapy alone, I-125 combined with chemotherapy, or I-125 combined with CHM, respectively. It was indicated that both chemotherapy and CHM, when combined with I-125 seed brachytherapy, significantly improve survival compared to I-125 brachytherapy alone. Specifically, the median overall survival (OS) was highest in the CHM group, followed by the chemotherapy group, and lowest in the Control group. Moreover, both chemotherapy and CHM significantly reduced mortality risk, with CHM offering a comparable survival benefit to chemotherapy. Additionally, CHM treatment demonstrated a lower incidence of adverse events compared to chemotherapy, particularly in reducing gastrointestinal toxicity and hematologic complications. These findings suggest that CHM may serve as an effective alternative or complementary treatment to chemotherapy in advanced NSCLC patients receiving I-125 seed brachytherapy.

The effectiveness of I-125 seed brachytherapy as a localized radiotherapy for NSCLC has been well documented in previous studies, demonstrating significant improvement in tumor control and survival outcomes compared to conventional external beam radiotherapy (EBRT) (15, 16). A meta-analysis by Zhang et al. (2) reported that I-125 seed brachytherapy combined with chemotherapy resulted in a 1-year survival rate of 86.5% compared to 57.6% for chemotherapy alone, which aligns with the improved survival observed in this study (5). Similarly, Huo et al. (17) found that I-125 implantation combined with trans-arterial chemotherapy infusion significantly prolonged progression-free survival (PFS) and overall survival (OS) compared to trans-arterial infusion alone, supporting the survival benefit observed in this study when chemotherapy was used alongside I-125 brachytherapy (17).

Our study also provides evidence for CHM as a potential alternative treatment to chemotherapy in combination with I-125 seed brachytherapy. Several studies have highlighted the therapeutic benefits of CHM in lung cancer, demonstrating its role in enhancing immune function, inducing tumor apoptosis, and reducing systemic toxicity (18, 19). A randomized controlled trial (RCT) conducted by Wang et al. (20) reported that CHM combined with maintenance chemotherapy significantly improved median OS and reduced chemotherapy-induced adverse effects compared to chemotherapy alone (7). In the realm of complementary and alternative medicine, CHM treatment is gaining increasing popularity among patients with advanced NSCLC (20, 21). The findings of our study reinforce these observations, as patients in the CHM group had comparable survival outcomes to those in the chemotherapy group but experienced fewer side effects and lower treatment costs.

The toxicity profile observed in our study is consistent with prior research, where chemotherapy was associated with significantly higher rates of leukopenia, thrombocytopenia, and anemia compared to CHM treatment. In contrast, CHM was associated with a lower incidence of adverse events, particularly in reducing nausea, vomiting, and hematologic toxicity, suggesting a better tolerability profile compared to chemotherapy (22). Moreover, our study showed that CHM was more cost-effective than chemotherapy, with treatment costs averaging 80.9 ± 18.2 thousand yuan for the CHM group compared to 95.8 ± 21.7 thousand yuan for the chemotherapy group. This aligns with studies demonstrating that CHM offers economic advantages by reducing hospitalizations and improving patient adherence to treatment (11, 13, 19, 23).

Despite the significant findings, this study has several limitations. First, as a retrospective, single-center study, this research is subject to potential selection bias. The absence of cytokine profiling, immune-related biomarkers, and pharmacokinetic profiles of Chinese herbal medicine (CHM) may limit the generalizability of the findings to broader populations. Future multicenter randomized controlled trials (RCTs) would provide more robust evidence to further validate the conclusions of this study. Second, the sample size for the CHM group was smaller than the chemotherapy and control groups, which may affect the statistical power of subgroup analyses, this increases the risk of Type II errors, and therefore, results should be interpreted with caution. Future studies with larger sample sizes are needed to validate these results. Third, while CHM has been shown to modulate immune function and exert anti-tumor effects, the exact molecular mechanisms remain unclear. Further biological and mechanistic studies are required to elucidate how CHM interacts with I-125 seed brachytherapy to enhance survival outcomes. Lastly, long-term side effects and quality-of-life assessments beyond the 5-year follow-up period were not examined in this study. Extended follow-up periods could provide insights into late toxicities and long-term survivorship outcomes.

## Conclusion

Collectively, the present study demonstrates that both chemotherapy and CHM, when combined with I-125 seed brachytherapy, significantly improve survival outcomes compared to I-125 brachytherapy alone. CHM provided a comparable survival benefit to chemotherapy while exhibiting a more favorable safety profile and lower treatment costs, making it a potential alternative or complementary therapy for NSCLC patients who cannot tolerate chemotherapy. These findings contribute to the growing body of evidence supporting integrative oncology approaches in lung cancer treatment. Future randomized, multicenter studies are needed to further explore the clinical efficacy, safety, and cost-effectiveness of CHM in NSCLC treatment.

## Data Availability

The raw data supporting the conclusions of this article will be made available by the authors, without undue reservation.
